# P050. Migrainous aura: when to worry?

**DOI:** 10.1186/1129-2377-16-S1-A85

**Published:** 2015-09-28

**Authors:** Matteo Fuccaro, Lucia Nardetto, Giorgio Zanchin, Federico Mainardi, Ferdinando Maggioni

**Affiliations:** Department of Neurosciences, Headache Centre, University of Padua, Padua, Italy; Department of Neurology, Hospital S. Antonio, Padua, Italy; Department of Neurology, Headache Centre, Hospital SS. Giovanni and Paolo, Venice, Italy

## Introduction

Migraine with aura (MA) is a common disorder affecting about 30% of migrainous patients[1]. MA is codified in the ICHD-III classification[2] and typical auras are known and easily recognized in particular by patients chronically suffering of MA. Sometimes even these patients present to the Emergency Department (ED) because their auras are different from usual. The more frequent causes are abnormal prolongation, increased frequency of recurrence or the developing of new symptoms, particularly in patients that presented in the past only visual auras. Generally these conditions are not dangerous, but ambiguous situations may occur, with the possibility of considering as trivial a potentially serious disorder, as in our patient.

## Case report

A 41-year old male suffered since his youth of sporadic episodes of migrainous headache preceded, 3-6 times/year, by typical auras consisting of a spreading right-sided visual field associated, after about ten minutes from the onset, with paresthesias ascending along the right arm. In 2010 he referred a transitory increase in frequency of these episodes and the MRI performed was normal. In November 2012 he experienced a transitory increase in frequency of the episodes, but also noticed an extension of the ascending paresthesia to his right leg. Duration of aura slightly increased, and was followed by “usual” headache. Evaluated in the ED, a brain-CT (b-CT) scan demonstrated the presence of a cortical-subcortical hemorrhage in the left posterior parietal lobe. During hospitalization, EEG and four vessel cerebral angiographies were normal. In May 2013 after a 6-month free-of-episodes period, he went again to the ED for several daily episodes of visual and paresthetic aura, involving the right arm; b-CT and MRI were negative for acute lesions; EEG was normal. Discharged with topiramate 50 mg/day, the patient presented in the following years only sporadic episodes of visual aura but in March 2015 he experienced again a recrudescence of daily episodes of aura. Admitted to our Neurology Ward, b-CT scan, EEG and MRI were unremarkable for new events.

## Discussion

In the ICHD-III classification[2] we find a group of MA complications as persistent aura without infarction and migrainous infarction; we performed a literature research to find if there were other possible modifications in typical aura presentation that could represent a risk factor of a complication as in the case here reported. The data about this topic are lacking and we think that prospective studies on this matter are desirable.

Written informed consent to publication was obtained from the patient(s).

## References

[1] Rasmussen BK, Olesen J (1992). Migraine with aura and migraine without aura: an epidemiological study. Cephalalgia.

[2] Headache Classification Committee of the International Headache Society (2013). The international classification of headache disorders: 3^th^ edition (beta version). Cephalalgia.

